# Predictors of acute kidney injury in dengue patients: a systematic review and meta-analysis

**DOI:** 10.1186/s12985-024-02488-7

**Published:** 2024-09-19

**Authors:** Abdelaziz A. Awad, Mahalaqua Nazli Khatib, Abhay M Gaidhane, Suhas Ballal, Pooja Bansal, Manish Srivastava, Isha Arora, M Ravi Kumar, Aashna Sinha, Pramod Rawat, Sanjit Sah, Ganesh Bushi, Sorabh Lakhanpal, Muhammed Shabil

**Affiliations:** 1 Faculty of Medicine, Azhar university, Cairo, Egypt; 2 Division of Evidence Synthesis, Global Consortium of Public Health and Research, Datta Meghe Institute of Higher Education, Wardha, India; 3 Jawaharlal Nehru Medical College, and Global Health Academy, School of Epidemiology and Public Health, Datta Meghe Institute of Higher Education, Wardha, India; 4 Department of Chemistry and Biochemistry, School of Sciences, JAIN (Deemed to be University), Bangalore, Karnataka India; 5 Department of Allied Healthcare and Sciences, Vivekananda Global University, Jaipur, 303012 Rajasthan India; 6 Department of Endocrinology, NIMS University, Jaipur, India; 7 Chandigarh Pharmacy College, Chandigarh Group of College, Jhanjeri, Mohali, 140307 Punjab India; 8 Department of Chemistry, Raghu Engineering College, Visakhapatnam, 531162 Andhra Pradesh India; 9 School of Applied and Life Sciences, Division of Research and Innovation, Uttaranchal University, Dehradun, India; 10 Department of Biotechnology, Graphic Era (Deemed to be University), Clement Town, Dehradun, 248002 India; 11 Department of Allied Sciences, Graphic Era Hill University Clement Town, Dehradun, 248002 India; 12 Department of Paediatrics, Hospital and Research Centre, Dr. D. Y. Patil Medical College, Dr. D. Y. Patil Vidyapeeth, Pune, 411018 Maharashtra India; 13 Department of Public Health Dentistry, Dr. D.Y. Patil Dental College and Hospital, Dr. D.Y. Patil Vidyapeeth, Pune, 411018 Maharashtra India; 14 Center for Global Health Research, Saveetha Institute of Medical and Technical Sciences, Saveetha Medical College and Hospital, Saveetha University, Chennai, India; 15 School of Pharmaceutical Sciences, Lovely Professional University, Phagwara, India; 16 University Center for Research and Development, Chandigarh University, Mohali, Punjab India; 17 Medical Laboratories Techniques Department, AL-Mustaqbal University, Hillah, Babil, 51001 Iraq

**Keywords:** Acute kidney injuries, Severe dengue, Systematic review, Public health, Dengue Virus, Kidney diseases

## Abstract

**Background:**

Dengue infection poses a significant global health challenge, particularly in tropical and subtropical regions. Among its severe complications, Acute kidney injury (AKI) stands out due to its association with increased morbidity, mortality, and healthcare burdens. This Meta-analysis aim to identify and evaluate the predictors of AKI among dengue patients, facilitating early detection and management strategies to mitigate AKI’s impact.

**Methods:**

We searched PubMed, EMBASE, and Web of Science databases, covering literature up to February 2024. We included human observational studies reporting on AKI predictors in confirmed dengue cases. Nested-Knowledge software was used for screening and data extraction. The Newcastle-Ottawa Scale was used for quality assessment. R software (V 4.3) was utilized to compute pooled odds ratios (ORs) and 95% confidence intervals (CIs) for each predictor.

**Results:**

Our search yielded nine studies involving diverse geographic locations and patient demographics. A total of 9,198 patients were included in the studies, with 542 diagnosed with AKI. in which key predictors of AKI identified include severe forms of dengue (OR: 2.22, 95% CI: 1.02–3.42), male gender (OR: 3.13, 95% CI: 1.82–4.44), comorbidities such as diabetes mellitus (OR: 3.298, 95% CI: 0.274–6.322), and chronic kidney disease (OR: 2.2, 95% CI: 0.42–11.24), as well as co-infections and clinical manifestations like rhabdomyolysis and major bleeding.

**Conclusion:**

Our study identifies several predictors of AKI in dengue patients. These findings indicate the importance of early identification and intervention for high-risk individuals. Future research should focus on standardizing AKI diagnostic criteria within the dengue context and exploring the mechanisms underlying these associations to improve patient care and outcomes.

**Supplementary Information:**

The online version contains supplementary material available at 10.1186/s12985-024-02488-7.

## Introduction

Dengue represents a significant global public health challenge, especially in tropical and subtropical regions, affecting millions yearly [1–3]. It manifests in a spectrum ranging from mild, flu-like symptoms to severe conditions, including dengue hemorrhagic fever, severe dengue, and dengue shock syndrome [1]. A particularly concerning complication of dengue is acute kidney injury (AKI), associated with increased morbidity, extended hospital stays, and elevated mortality rates [4–6]. The mechanisms predisposing individuals to AKI during an infection with dengue virus have yet to be fully understood, complicating early detection and effective management.

The reported prevalence of AKI among those with dengue varies widely [5]. This variation is influenced by several factors, including geographic differences, the specific serotypes of the dengue virus present, demographic factors of the populations studied, and the criteria used to define AKI clinically [7]. For example, regions with limited healthcare resources might underreport AKI cases due to a scarcity of diagnostic capabilities or a lack of healthcare provider awareness [8, 9]. Moreover, certain dengue virus serotypes have been linked to more severe disease manifestations and, consequently, an increased risk of AKI [10, 11]. Identifying AKI predictors in the context of dengue is crucial for developing strategies that can effectively detect, categorize risks, and intervene early to minimize the incidence and impact of AKI. Demographic factors such as age, gender, and existing comorbidities significantly affect the risk of developing AKI in dengue patients. Older individuals and those with underlying conditions, like diabetes, hypertension, or chronic kidney disease, are particularly at high risk to AKI during dengue infection [12]. Additionally, studies suggest that males might have a higher risk of severe dengue and subsequent AKI, indicating a complex interaction of genetic, hormonal, and environmental factors [11, 13, 14]. The use of varied criteria across studies to define AKI, including the Risk, Injury, Failure, Loss of kidney function, and End-stage kidney disease (RIFLE) criteria, the Acute Kidney Injury Network (AKIN) criteria, or the Kidney Disease: Improving Global Outcomes (KDIGO) criteria, complicates the understanding of the true prevalence of AKI in dengue infections [15]. These inconsistencies highlight the need for standardized diagnostic and reporting protocols for AKI in the context of dengue, facilitating more accurate study comparisons and analyses.

Understanding the factors influencing AKI development in dengue patients is critical, as it aids in the early identification of AKI and facilitates timely intervention. This systematic review seeks to elucidate the predictors of AKI in individuals infected with dengue, providing a foundation for improved patient care and outcomes.

## Methods

The systematic review and meta-analysis were conducted in adherence to the Preferred Reporting Items for Systematic Reviews and Meta-Analyses (PRISMA) guidelines [16], as detailed in Table S1. The review protocol was officially registered in the Prospero database under the registration number: CRD42024517289. Nested knowledge web software (Nested-Knowledge, MN, USA) was used in this study for screening of articles and data extraction.

### Literature search strategy

A comprehensive search was performed across three electronic databases: PubMed, EMBASE, and Web of Science. The search covered records from their inceptions until December 20, 2023, with an update on February 15, 2024. The search strategy employed keywords and MeSH terms. A detailed description of the search strategy is available in Table S2. Searches were conducted without any restrictions based on language, publication type, or publication year.

### Inclusion and exclusion criteria

Inclusion criteria were set to human observational studies, such as cross-sectional, case-control, or cohort studies, that reported on AKI and its predictors among dengue patients. No restrictions were imposed on the demographics of the eligible population. Only confirmed dengue cases were considered. Exclusion criteria were case reports, case series, review articles, letters, in vitro and animal studies, conference abstracts, and studies that did not distinguish between severe and non-severe dengue outcomes. Only studies published in English with clearly stated criteria for AKI diagnosis were included .

### Study selection

The articles retrieved from databases were imported into Nested Knowledge web software for initial screening, where duplicates were removed. Titles and abstracts were preliminarily reviewed, followed by full-text screening to determine final inclusion. Two independent reviewers carried out this process, with any disagreements resolved through discussion or consulting a third reviewer.

### Data extraction and quality evaluation

Data extraction was performed by two reviewers (SB and MS), with any differences resolved by mutual consensus. Extracted data included the first author’s name, country of study, study design, sample size, study population demographics, median or mean age, percentage of male participants, predictors of AKI, and their associated estimates. We used the “tagging” function of Nested Knowledge software to aid data extraction. The Newcastle-Ottawa Scale (NOS) was utilized to assess the quality of the included studies. Reviewers (SB and MS) conducted this quality assessment independently, with any discrepancies resolved through consensus or the involvement of an additional author (MNK).

### Statistical analysis

In our analysis, we employed a random-effects model to compute the pooled odds ratios (ORs) and 95% confidence intervals (CIs) for each identified predictor of AKI, specifically employing the Restricted Maximum Likelihood (REML) method for estimating between-study variance [17]. This model was selected to better accommodate the expected variability across studies due to population, design, and methodology differences, thus providing a more accurate estimation of the true effect size. To evaluate the heterogeneity among the included studies, we utilized tau-square and I^2^ statistics, categorizing heterogeneity levels as low (< 25%), low to moderate (25% to < 50%), moderate to high (50% to < 75%), or high (≥ 75%) [18, 19]. These categories help to understand the variance among study outcomes and the generalizability of our findings. All statistical analyses were performed using R software, version 4.3 [20].

## Results

### Literature search and study characteristics

**Fig. 1:**
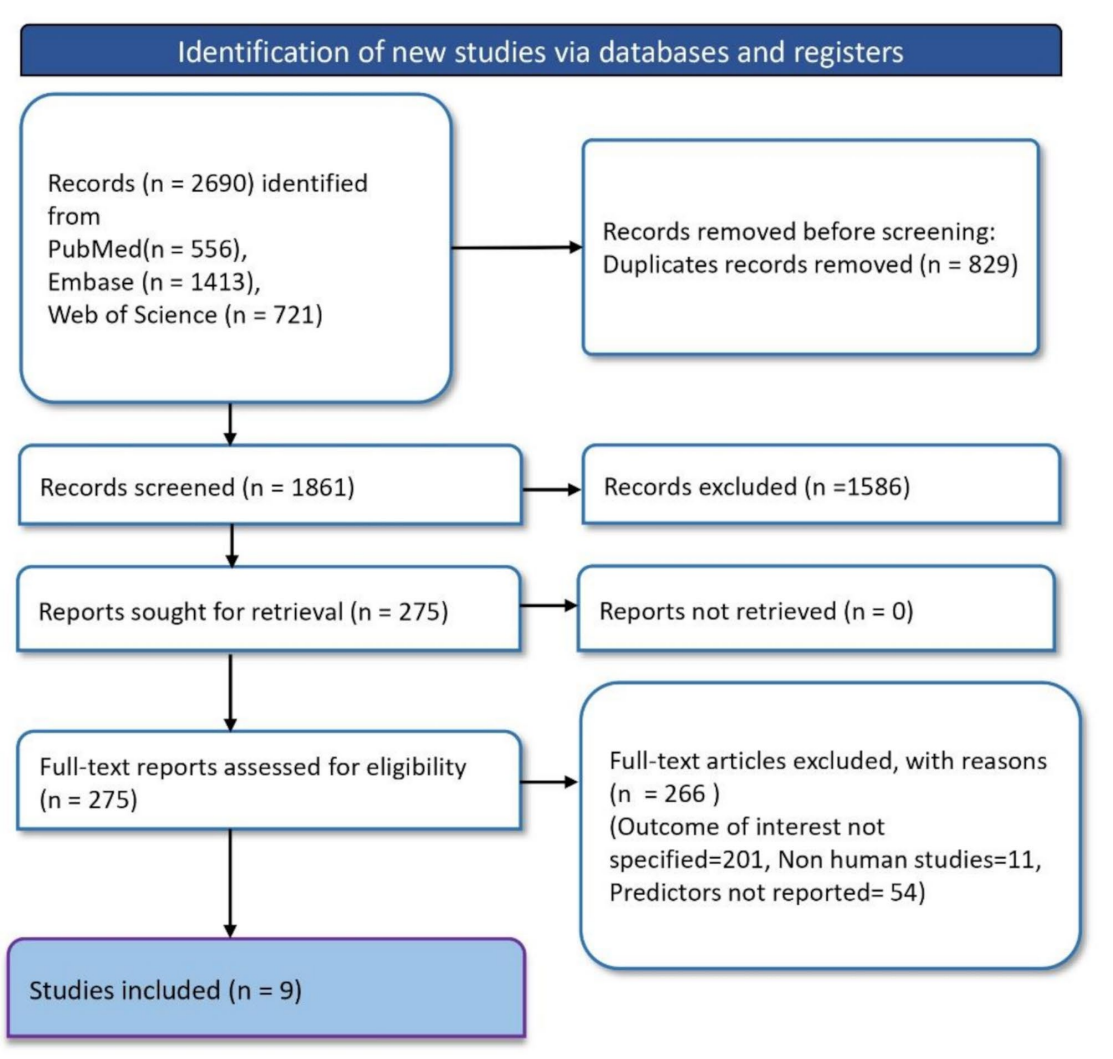
PRISMA flowchart depicting article selection and screening process

The search for literature in the systematic review commenced with identifying 2,690 records through various databases: 556 from PubMed, 1,413 from Embase, and 721 from Web of Science. Before screening, duplicates were meticulously removed, totalling 829 records. The screening process was then conducted on 1,861 records. No reports were excluded as not retrievable, resulting in 275 reports sought for full-text retrieval. Upon a thorough assessment of these full texts for eligibility, 266 articles were excluded for the following reasons: 201 due to the outcome of interest not being specified, 11 for being non-human studies, and 54 for not reporting the predictors of interest. Ultimately, nine studies were included in the review (Fig. 1).

The characteristics of the included studies are presented in Table 1. Two of the nine studies were from India [11, 21], two from Thailand [14, 22] while other studies conducted in Indonesia [23], Vietnam [13], Pakistan [24], Malaysia [4], and China [7]. A total of 9,198 patients were included in the studies, with 542 diagnosed with AKI. Most of these studies employed a retrospective design, indicative of the common approach to analyzing clinical outcomes in existing patient data, except for a single cross-sectional study from Vietnam, which provided insights into the prevalence of AKI among hospitalized dengue patients at a particular time. Participant demographics across the studies showed a wide age range, from children under 15 to elderly patients with a mean age of 70, and a varied gender distribution, with male participants comprising 47.4–70.9% of the study populations. Diagnostic methods for confirming dengue infection included NS1 antigen detection, IgM/IgG antibodies by ELISA, and PCR for viral RNA, reflecting standard practices for accurate dengue diagnosis. The criteria for determining AKI among the studies were based on established clinical guidelines such as KDIGO, AKIN, and RIFLE, which rely on changes in serum creatinine levels and urine output. The quality of the studies is given in Table S4.

**Table 1:** Characteristics of included studies

Study	Country	Design	Participants	Total sample	Mean/Median age in years	Male %	Number of patients with AKI	AKI criteria	Dengue diagnosis	Time period
Diptyanusa 2019 (23)	Indonesia	Retrospective study	Hospitalized ≥ 18 years old dengue patients	1,484	28 (IQR: 22 to 40)	50.4	71	KDIGO	NS1 result, presence of IgM/IgG antibodies in acute phase serum (ELISA), or PCR	January 2012 to November 2017
Huy 2020 (13)	Vietnam	Cross-sectional	Hospitalized patients with DVI aged > 18 years	2,417	33.4 (SD: ± 12.7)	47.4	64	SCr increase ≥ 0.3 mg/dL within 48 h or SCr rise to 1.5 times baseline in the last 7 days	NS1 or IgM/IgG or PCR method	January to December 2017
Khalil 2012 (24)	Pakistan	Retrospective study	Patients aged > 14 years and hospitalized	532	35.29 (SD: ± 14.7)	70.9	71	AKIN	IgM anti-bodies	January 2008 to December 2010
Laoprasopwattana 2010 (22)	Thailand	Retrospective study	Children aged < 15 years with DVI-induced AKI	2893	9.1 (SD: ± 3.6)	56	25	SCr levels > 2 mg/dL or a SCr concentration > 2 times previous or subsequent values	IgM and HAI	January 1989 to December 2007
Mallhi 2015 (4)	Malaysia	Retrospective study	Hospitalized patients with DVI aged > 12 years	667	30.68 (SD: ± 16.13)	56.7	95	AKIN	RT-PCR, IgM/IgG (ELISA), ≥ 4-fold increase in HAI titers in convalescent serum, and NS1 antigen	January 2008 to December 2013
Mehra 2012 (21)	India	Retrospective study	Inpatients with dengue fever	223	26.2 (SD:18.2)	58.3	24	AKIN	ELISA for IgM antibody in acute phase serum	NA
Patel 2019 (11)	India	Retrospective study	Inpatients > 14 years of age	620	Male = 31.6 (± 12.8), female = 29.6 (SD: ±11.2)	56.93	90	AKIN	RT-PCR and the presence of dengue IgM and IgG antibodies in acute phase serum (ELISA)	January 2016 to December 2017
Surasombatpattana 2021(14)	Thailand	Prospective study	Hospitalized patients with DVI aged > 18 years	120	42 (IQR: (23 to 50)	63	17	RIFLE	NS1 antigen, viral PCR, seroconversion of IgG antibodies (≥ 4-fold rise), or IgM antibodies in acute phase serum	January 2017 to December 2019
Wang 2023 (7)	China	Retrospective study	Patients over 18 years with severe dengue	242	70 (51 to 79)	57	85	KDIGO	NS1 antigen (ELISA), DENV RNA (RT-PCR), and/or IgG antibody seroconversion on ELISA	January 2013 and November 2019

### Predictors of AKI in dengue

**Fig. 2:**
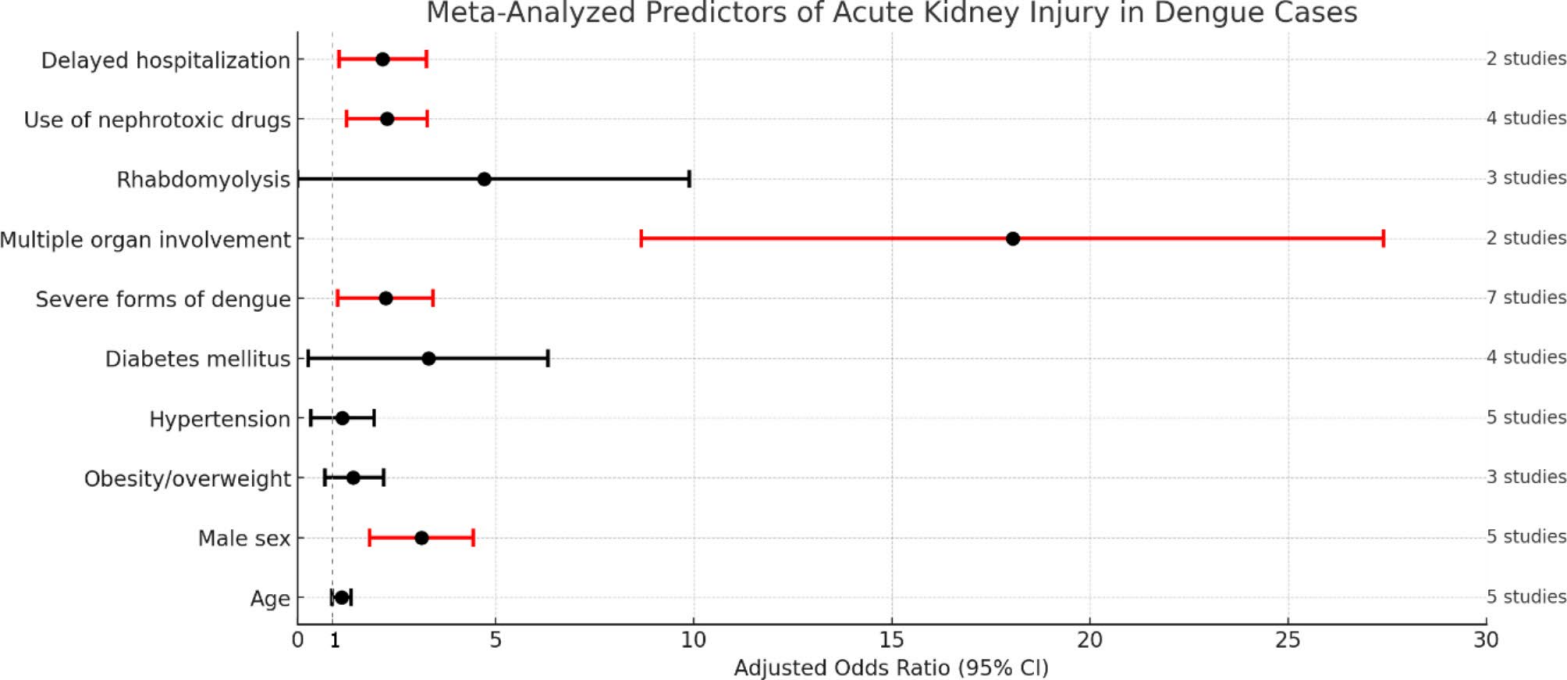
Predictors of Acute kidney injury among dengue cases based on meta-analysis of multiple studies. Red line indicates effect size and CI of significant predictors. Black line indicates effect size and CI of non-significant predictors. Dotted vertical line indicate point of no effect

**Fig. 3:**
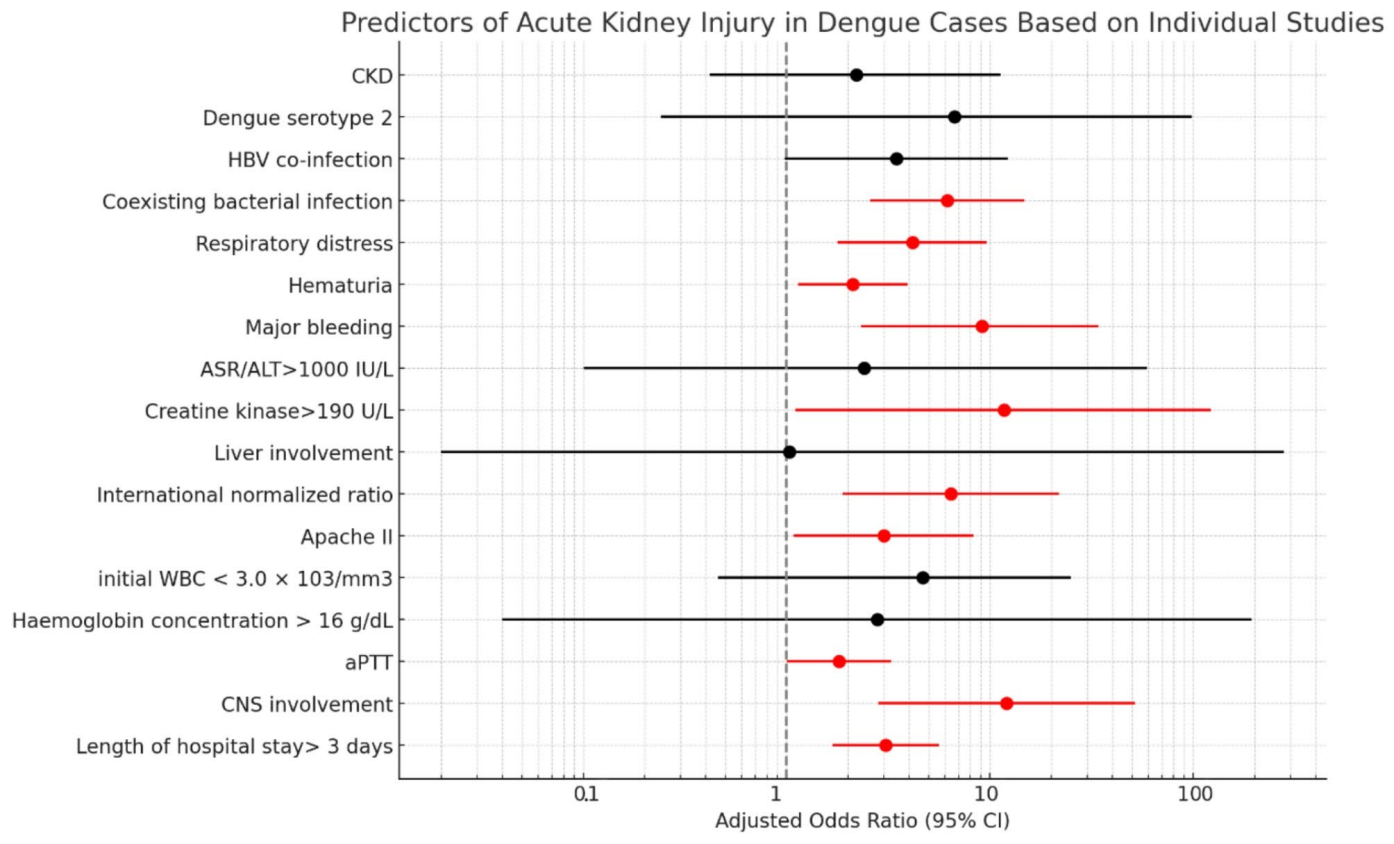
Predictors of Acute kidney injury among dengue cases reported by individual studies. Red line indicates effect size and CI of significant predictors. Black line indicates effect size and CI of non-significant predictors. Dotted vertical line indicate point of no effect. Abbreviations: aPTT - Activated Partial Thromboplastin Time, ASR/ALT - Aspartate Aminotransferase/Alanine Aminotransferase, CKD - Chronic Kidney Disease, CNS - Central Nervous System, HBV - Hepatitis B Virus, WBC - White Blood Cell

The predictors of AKI in dengue infection identified through meta-analysis are summarized in Table 2 and illustrated in Fig. 2. Predictors identified from single studies are presented separately in Table 3; Fig. 3.

**Table 2:** Meta-analyzed predictors of acute kidney injury among dengue cases

Predictors of AKI	Number of studies	Adjusted OR (95% CI)	Heterogeneity (I^2^)
**Demographics**			
Age	5	1.11 (0.87–1.35)	0%
Male sex	5	3.13 (1.82–4.44)	0%
Obesity/overweight	3	1.4 (0.69–2.18)	0%
**Comorbidities**			
Hypertension	5	1.13 (0.34–1.93)	0%
Diabetes mellitus	4	3.298 (0.274–6.322)	0%
**Dengue related**			
Severe forms of dengue	7	2.22 (1.02–3.42)	8%
**Clinical manifestations**			
Multiple organ involvement	2	18.04 (8.68–27.4)	0%
Rhabdomyolysis	3	4.71 (0.0-9.89)	44%
**Others**			
Use of nephrotoxic drugs	4	2.26 (1.24–3.27)	0%
Delayed hospitalization	2	2.14 (1.04–3.25)	0%

**Table 3:** Single-study estimates of predictors of acute kidney injury among dengue cases

Predictors of AKI	Adjusted OR (95% CI)
**Comorbidities**	
CKD	2.2 (0.42–11.24)
**Dengue related**	
Dengue serotype 2	6.67 (0.24–98.12)
**Co-infections**	
HBV co-infection	3.46(0.98–12.23)
Coexisting bacterial infection	6.15 (2.57–14.74)
**Clinical manifestations**	
Respiratory distress	4.15 (1.79–9.63)
Hematuria	2.12 (1.14–3.95)
Major bleeding	9.12 (2.33–34.12)
ASR/ALT > 1000 IU/L	2.4 (0.1–59.3)
Creatine kinase > 190 U/L	11.7 (1.1-122.4)
Liver involvement	1.03 (0.02-279.37)
International normalized ratio	6.44 (1.89–21.95)
Apache II	3.00 (1.08–8.33)
initial WBC < 3.0 × 103/mm3,	4.67 (0.46-25.00)
Haemoglobin concentration > 16 g/dL	2.8 (0.04-193.87)
aPTT	1.81 (1.003–3.26)
CNS involvement	12.08 (2.82–51.77)
**Others**	
Length of hospital stay > 3 days	3.07 (1.68–5.62)

### Demographics

Age was evaluated in five studies, and the analysis yielded an adjusted OR of 1.11 (95% CI: 0.87–1.35), indicating a slight but not statistically significant increase in the risk of AKI with advancing age. The heterogeneity among these studies was reported as 0%, suggesting high consistency in the findings across different research settings. The factor of male sex was also examined in five studies, revealing a more pronounced association with AKI. The adjusted OR was 3.13 (95% CI: 1.82–4.44), indicating that males with dengue are over three times more likely to develop AKI than females, with 0% heterogeneity, underscoring the robustness and reliability of the association across different studies. Obesity or overweight status was explored in three studies as a potential predictor of AKI in dengue patients. The analysis yielded an adjusted OR of 1.4 (95% CI: 0.69–2.18), which suggests a moderate increase in the risk of AKI among obese or overweight individuals. However, this association did not reach statistical significance. Similar to the other demographic factors, the heterogeneity for this predictor was reported as 0%, indicating consistent findings across the included studies.

### Comorbidities

Hypertension was assessed in five studies, with the findings indicating an OR of 1.13 (95% CI: 0.34–1.93). This suggests a marginally increased risk of AKI among dengue patients with hypertension, although the wide confidence interval indicates a degree of uncertainty in the exact magnitude of this risk. The heterogeneity for this association was reported as 0%. Diabetes mellitus was analyzed in four studies, revealing a association with AKI in dengue patients. The adjusted OR for this comorbidity was 3.298 (95% CI: 0.274–6.322), indicating that individuals with diabetes mellitus are significantly more likely to develop AKI when suffering from dengue. Despite the wide confidence interval, which suggests variability in the effect size, 0% heterogeneity was noted. Chronic kidney disease (CKD) was examined in a single study, which identified it as a potential risk factor for AKI in dengue patients. The study reported an adjusted OR of 2.2 (95% CI: 0.42–11.24). Although this finding is based on a limited number of studies and the confidence interval is wide, suggesting a high degree of uncertainty, it does indicate a possible increased risk of AKI for dengue patients with pre-existing CKD.

### Dengue severity and serotype

Severe forms of dengue, such as DHF and severe dengue, were analyzed across seven studies, highlighting a significant association with an increased risk of AKI. The combined data yielded an OR of 2.22 (95% CI: 1.02–3.42). This indicates that individuals suffering from severe dengue, characterized by more critical symptoms and complications, are over two times more likely to develop AKI compared to those with less severe forms of the disease. The heterogeneity among these studies was remarkably low, at 8%, suggesting a relatively consistent association across different research settings and populations. The analysis of dengue serotype 2 as a specific risk factor for AKI was conducted in a single study, which found high adjusted OR of 6.67 (95% CI: 0.24–98.12). Despite the broad confidence interval, this result indicates a potentially substantial increase in the risk of AKI associated with dengue serotype 2. However, the wide confidence interval and the fact that this finding is based on a single study underscore the need for caution in interpretation and highlight the importance of further research to confirm the role of dengue serotype 2 in the development of AKI.

### Co-infections

The role of co-infections in exacerbating the risk of AKI in dengue patients has been explored through the analysis of hepatitis B virus (HBV) co-infection and coexisting bacterial infections. The presence of these co-infections alongside dengue virus infection appears to significantly influence the likelihood of developing AKI, highlighting the complexity of managing patients with multiple concurrent infections. The analysis of HBV co-infection, conducted in a single study, indicated a notable increase in the risk of AKI among dengue patients. The adjusted OR for this co-infection was 3.46 (95% CI: 0.98–12.23). Although the confidence interval suggests some uncertainty in the magnitude of the effect, the point estimate indicates that dengue patients with HBV co-infection are over three times more likely to develop AKI compared to those without HBV co-infection. Coexisting bacterial infection was also identified as a significant risk factor for AKI in dengue patients, based on a single study. The adjusted OR for AKI in the presence of a bacterial infection was strikingly high at 6.15 (95% CI: 2.57–14.74).

### Clinical manifestations

Multiple organ involvement has been identified as a significant predictor of AKI, with an OR of 18.04 (95% CI: 8.68–27.4) based on two studies. This indicates a dramatically increased risk of AKI in the presence of multi-organ dysfunction, emphasizing the severity of dengue’s impact on patients. The heterogeneity for this predictor was reported as 0%, indicating consistent findings across studies. Rhabdomyolysis, analyzed in three studies, showed an OR of 4.71 (95% CI: 0.0-9.89), with a heterogeneity of 44%. This suggests a notable association between rhabdomyolysis and AKI risk, although the wide confidence interval indicates uncertainty in the precise effect size. Respiratory distress and hematuria were each analyzed in single studies, showing ORs of 4.15 (95% CI: 1.79–9.63) and 2.12 (95% CI: 1.14–3.95), respectively. These findings suggest that both respiratory distress and hematuria are associated with an increased risk of AKI in dengue patients. Major bleeding was another significant predictor, with a single study reporting an OR of 9.12 (95% CI: 2.33–34.12), indicating a substantial increase in AKI risk in the context of severe bleeding events. Extremely elevated liver enzymes, indicated by ASR/ALT levels greater than 1000 IU/L, were reported to potentially double the risk of AKI, with an adjusted OR of 2.4, although the wide confidence interval of 0.1–59.3 signals significant uncertainty in this estimate. Similarly, elevated creatine kinase levels above 190 U/L, indicative of muscle breakdown, were linked to a substantially increased risk of AKI, with an OR of 11.7 (95% CI: 1.1-122.4), reflecting considerable variability. Direct evidence of liver involvement in dengue showed a marginal association with AKI risk (OR of 1.03), but the extremely broad confidence interval (0.02-279.37) highlights the uncertainty surrounding this association. An elevated International Normalized Ratio (INR), signaling coagulation abnormalities, was associated with a significant increase in AKI risk (OR of 6.44), suggesting that patients with coagulopathy are at heightened risk. The Apache II score, a measure of disease severity, also indicated that higher severity scores correlate with increased AKI risk (OR of 3.00), emphasizing the link between disease severity and kidney injury. Low white blood cell counts, specifically initial WBC less than 3.0 × 10^3/mm^3, suggested an increased risk of AKI (OR of 4.67), pointing to the impact of significant leukopenia on kidney health. High hemoglobin concentrations, potentially indicative of hemoconcentration, showed a possible but uncertain increase in AKI risk (OR of 2.8), while prolonged activated partial thromboplastin time (aPTT) was mildly associated with an increased risk of AKI (OR of 1.81). Central nervous system involvement in dengue was linked to a significant increase in the risk of developing AKI, with an OR of 12.08, highlighting the severe implications of CNS complications.

### Nephrotoxic drugs

The use of nephrotoxic drugs among dengue patients has been identified as a significant factor associated with an increased risk of AKI, based on an analysis of four studies. The combined data from these studies yielded an adjusted OR of 2.26 (95% CI: 1.24 – 3.27). This finding indicates that patients with dengue who are exposed to nephrotoxic drugs are more than twice as likely to develop AKI compared to those not exposed to such medications.

### Delayed hospitalization and length of hospital stay

Delayed hospitalization, analyzed in two studies, showed an adjusted OR of 2.14 with a 95% CI ranging from 1.04 to 3.25. This indicates that patients with dengue who experience delays in receiving hospital care are over twice as likely to develop AKI compared to those who are hospitalized promptly. The implication is clear: timely access to medical care is crucial for minimizing the risk of severe complications such as AKI in dengue infection. The length of hospital stay specifically stays longer than three days, was examined in a single study and found to be associated with an increased risk of AKI, with an OR of 3.07 (CI: 1.68–5.62). This suggests that patients with dengue requiring prolonged hospitalization are significantly more likely to suffer from AKI, possibly reflecting the severity of their condition or complications arising during their care.

## Discussion

This is the first systematic review and meta-analysis to assess the predictors of AKI in dengue patients, highlighting critical insights into the complexity of managing dengue infections, particularly concerning AKI development. Our findings reveal a multifaceted interaction of demographic, clinical, and laboratory factors significantly influencing AKI risk among dengue patients. The analysis identified several key predictors and risk factors for AKI development, including CKD, severe forms of dengue, rhabdomyolysis, respiratory distress, hematuria, major bleeding, elevated ASR/ALT levels, liver involvement, an elevated International Normalized Ratio, Apache II scores, initial WBC, hemoglobin concentrations, prolonged aPTT, CNS involvement, and a length of hospital stay > 3 days. However, only a few studies were available for some of these predictors, and the populations varied across studies, with some focusing on children and others on adults. Similarly, the criteria used for AKI diagnosis varied among the studies. Due to the limited number of studies for some outcomes, we could not perform a publication bias analysis.

The association of AKI with demographic factors such as age and male gender, as well as with certain comorbidities including diabetes mellitus and chronic kidney disease, underscores the need for a targeted approach in the clinical management of dengue. Particularly, the strong association between male gender and AKI risk may reflect underlying biological differences or perhaps differences in exposure or health-seeking behavior between genders. The significant link between diabetes mellitus and AKI risk highlights the importance of managing metabolic conditions as part of comprehensive dengue care. the findings regarding the association of AKI with severe forms of dengue and certain clinical manifestations, such as rhabdomyolysis, respiratory distress, and major bleeding, emphasize the critical nature of these conditions and the necessity for early recognition and intervention. These factors not only serve as markers of disease severity but also as potential triggers for the development of AKI, suggesting that aggressive management of severe dengue may help mitigate the risk of AKI. The observed relationship between nephrotoxic drugs and AKI risk in dengue patients also warrants attention. This finding calls for cautious use of medications in the management of dengue, especially in settings where patients may already be at increased risk of kidney injury. Clinicians must balance the need for specific treatments against the potential harm, emphasizing the need for vigilant monitoring and adjustment of drug therapies in this patient population.

A previous systematic review assessed the prevalence of AKI in dengue patients, synthesizing data from 37 studies and involving 21,764 participants with dengue [5]. This analysis found an overall AKI prevalence of 8%. Focusing on gender, the review included findings from seven studies that reported data separately for male and female patients. It revealed that AKI was more prevalent among males (17%) compared to females (3%). The authors suggest that this disparity indicates a higher susceptibility to AKI in males, potentially due to the role of testosterone in promoting renal tubular cell inflammation and fibrosis, a mechanism that animal studies have supported.

The studies included in our analysis employed varying definitions of AKI, which may significantly impact the interpretation and generalizability of our findings. The criteria used across the studies included the RIFLE criteria, the AKIN criteria, and the KDIGO guidelines. Each of these frameworks has distinct thresholds for diagnosing AKI, based on changes in serum creatinine levels and urine output. This lack of uniformity in defining AKI could lead to discrepancies in the reported incidence and identified risk factors, making it difficult to compare results across studies and potentially introducing bias into the pooled estimates. Therefore, while our meta-analysis offers valuable insights into the predictors of AKI in dengue patients, the findings should be interpreted with caution.

Our review has several implications for clinical practice. It indicates the importance of early identification and aggressive management of dengue patients with high-risk profiles for AKI. This includes not only those with severe dengue but also patients presenting with specific risk factors such as advanced age, male gender, existing comorbidities like diabetes mellitus and CKD, and clinical manifestations indicative of multi-organ involvement. the findings highlight the need for clinicians to exercise caution when prescribing nephrotoxic drugs to dengue patients, advocating for the monitoring of renal function and the use of alternative treatments where feasible. Additionally, the association of delayed hospitalization with increased AKI risk emphasizes the importance of timely medical intervention, suggesting that public health measures should aim to reduce barriers to healthcare access and encourage early hospital presentation for dengue patients. This review identifies several areas for future research, including the need for studies that explore the mechanisms underlying the association between dengue infection and AKI, the role of genetic and environmental factors in influencing AKI risk, and the effectiveness of specific interventions to prevent AKI in this population. Furthermore, research should aim to standardize the diagnostic criteria for AKI in the context of dengue to facilitate more accurate assessments and comparisons across studies. our findings reinforce the need for strategies to prevent dengue infection and its complications, including vector control measures, public education campaigns, and the development of effective vaccines. Moreover, healthcare systems in dengue-endemic areas should be strengthened to improve the early detection and management of dengue and its complications, including AKI.

Integrating demographic, clinical, and laboratory data helps stratify patient risk and tailor interventions, aligning with the World Health Organization’s (WHO) sustainable development goal of healthy lives and promoting well-being for all ages by reducing disease and mortality rates [25]. Recognizing the complex contributors to AKI in dengue patients enables healthcare providers to enhance early detection, monitoring, and management strategies. Ultimately, these efforts aim to alleviate the burden of AKI on patients and healthcare systems.

Our study has several limitations. We only included published datasets, potentially overlooking relevant unpublished data or studies in the gray literature. Some predictors were reported in only a single study, limiting the ability to generalize findings across different populations and settings. We could not statistically assess the presence of publication bias due to the limited number of studies for some outcomes, which may influence the reliability of our results. The variables adjusted for in the calculation of ORs varied among the studies, introducing variability in risk estimates. There is heterogeneity among the populations studied, particularly in terms of age differences, which could affect the applicability of the findings to broader demographics. This heterogeneity underscores the need for cautious interpretation of our results and suggests that future research should aim to include a more diverse and comprehensive dataset to validate our findings and enhance our understanding of AKI predictors in dengue patients. Studies which evaluate the association of each of these factors are required in future.

## Conclusion

Our analysis provides crucial insights into the predictors of AKI among dengue patients, highlighting the multifaceted nature of risk factors ranging from demographic and clinical profiles to specific dengue virus serotypes and co-infections. The findings reinforce the importance of early identification and targeted management of high-risk individuals to mitigate the development of AKI, which can significantly worsen patient outcomes. Future research should focus on studying each of these factors and aim to standardize AKI diagnostic criteria within the context of dengue to enhance the accuracy and generalizability of the results.

## Electronic supplementary material

Below is the link to the electronic supplementary material.

Supplementary Material 1

## Data Availability

All data are presented within the manuscript and are available by contacting the corresponding author.
